# Supplementary material for: Development of the RF-GSEA Method for Identifying Disulfidptosis-Related Genes and Application in Hepatocellular Carcinoma
Source: Curr Issues Mol Biol. 2023 Nov 24;45(12):9450–70. doi: 10.3390/cimb45120593 (PMC10741996; doi:10.3390/cimb45120593)
Supplement: Supplementary file 1 [file cimb-45-00593-s001.zip › supplementary figures.pdf]

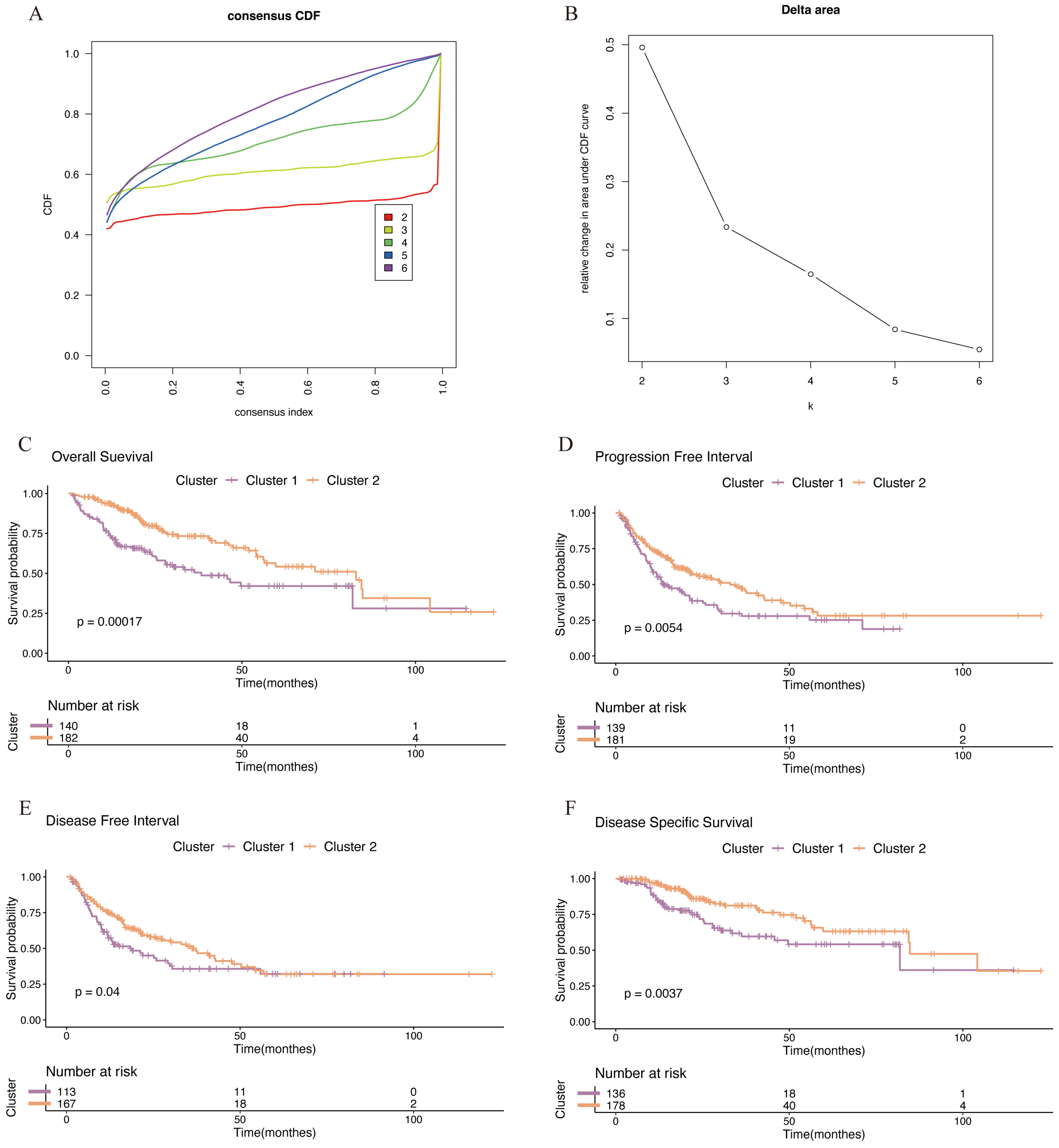

Figure S1. Consensus clustering analysis based on 84 disulfidptosis-related genes. (A) The area under the cumulative distribution function curve. (B) The Delta Area Plot. (C-F) Kaplan-Meier analysis for OS, PFI, DFI, and DSS in different clusters.

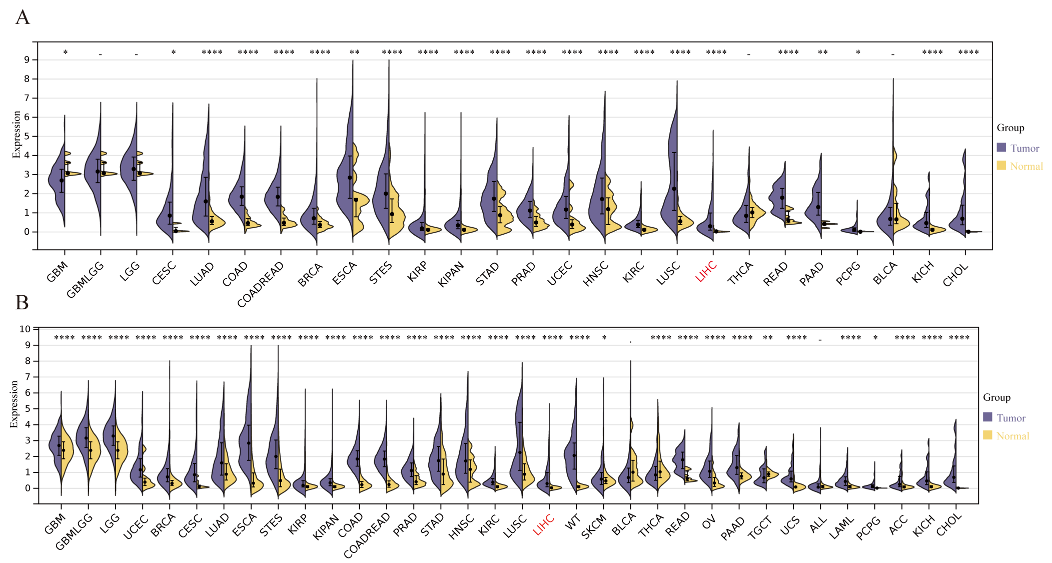

Figure S2. The expression level of SLC7A11 across different cancers. (A) Only TCGA database. (B) TCGA combine GTEx database.

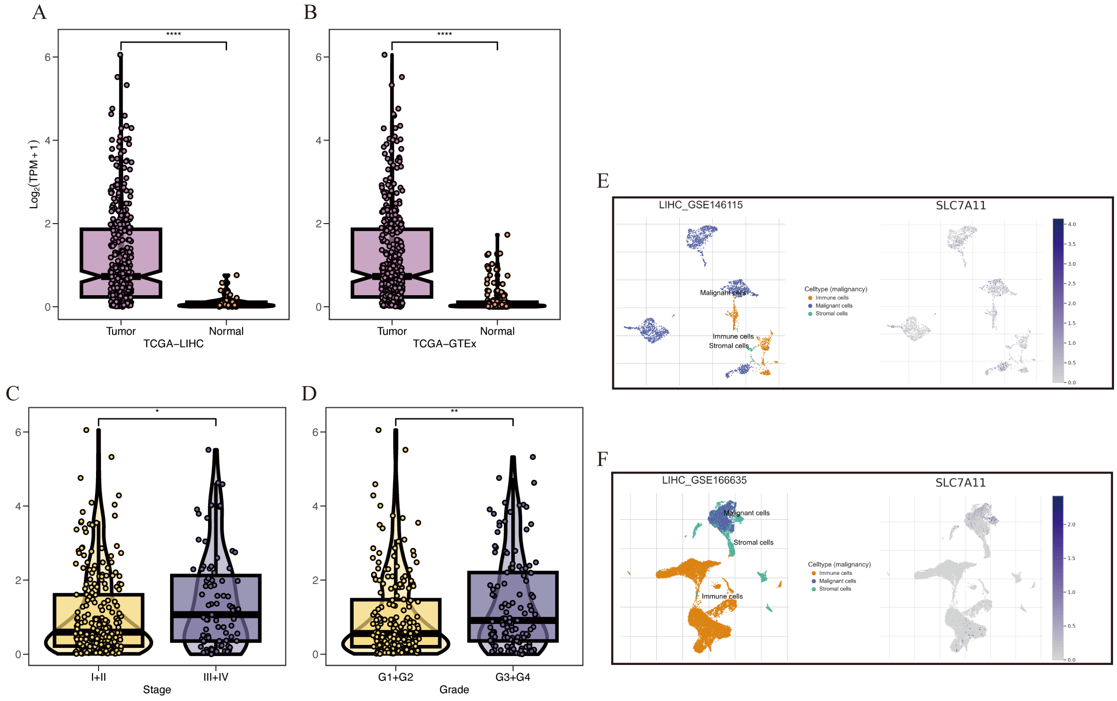

Figure S3. SLC7A11 expression pattern in HCC. (A-B) The expression levels of SLC7A11 in HCC tumor tissues and normal tissues in TCGA and TCGA combined with GTEx datasets. (C-D) The expression levels of SLC7A11 at different stages of HCC. (E-F) The expression of SLC7A11 in different cell types in HCC.

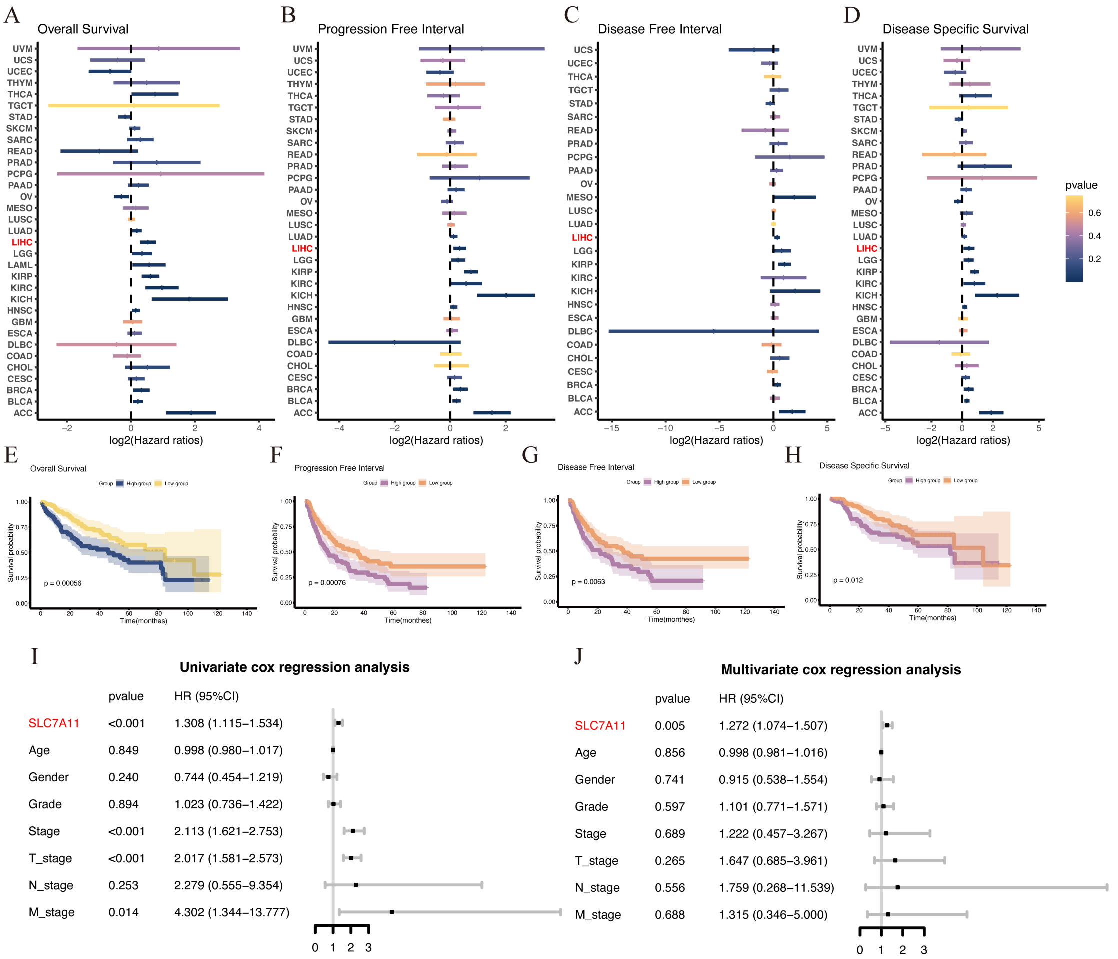

Figure S4. The prognostic value of SLC7A11 in cancers. (A-D) The correlation of SLC7A11 with OS, PFI, DFI, and DSS in various cancers. (E-H) K-M analysis of SLC7A11 in HCC. (I-J) Evaluating the prognostic value of SLC7A11 in HCC in comparison to clinical characteristics.

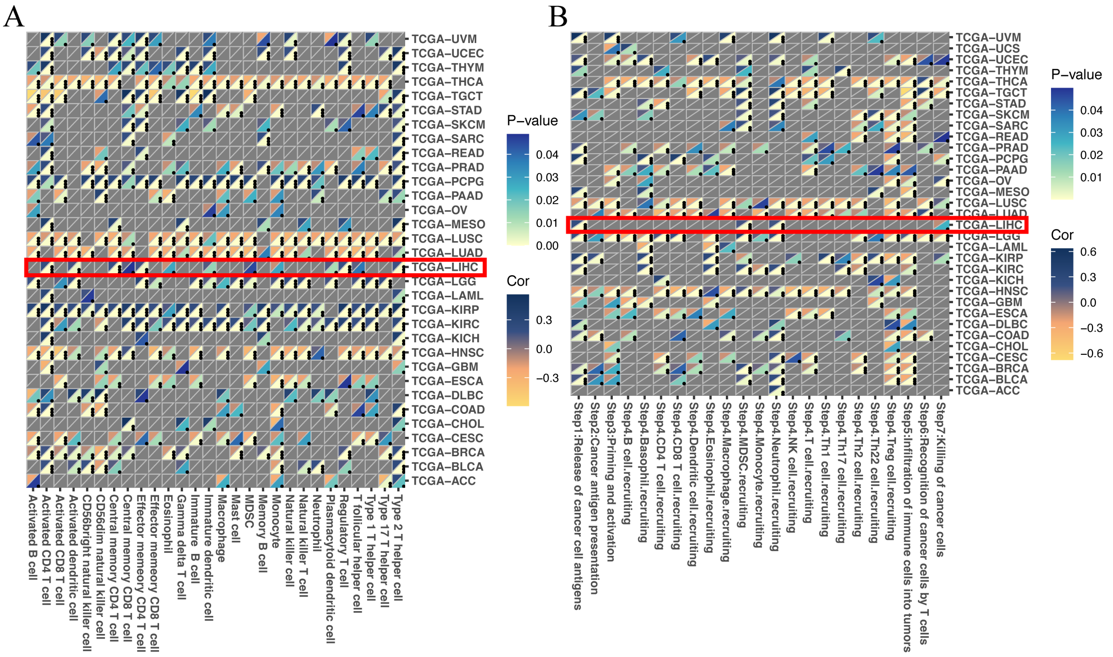

Figure S5. The correlation of SLC7A11 with the tumor microenvironment in various cancers. (A) The relationship between SLC7A11 and immune cell infiltration based on ssGSEA analysis. (B) The relationship between SLC7A11 and Cancer-Immunity Cycle.

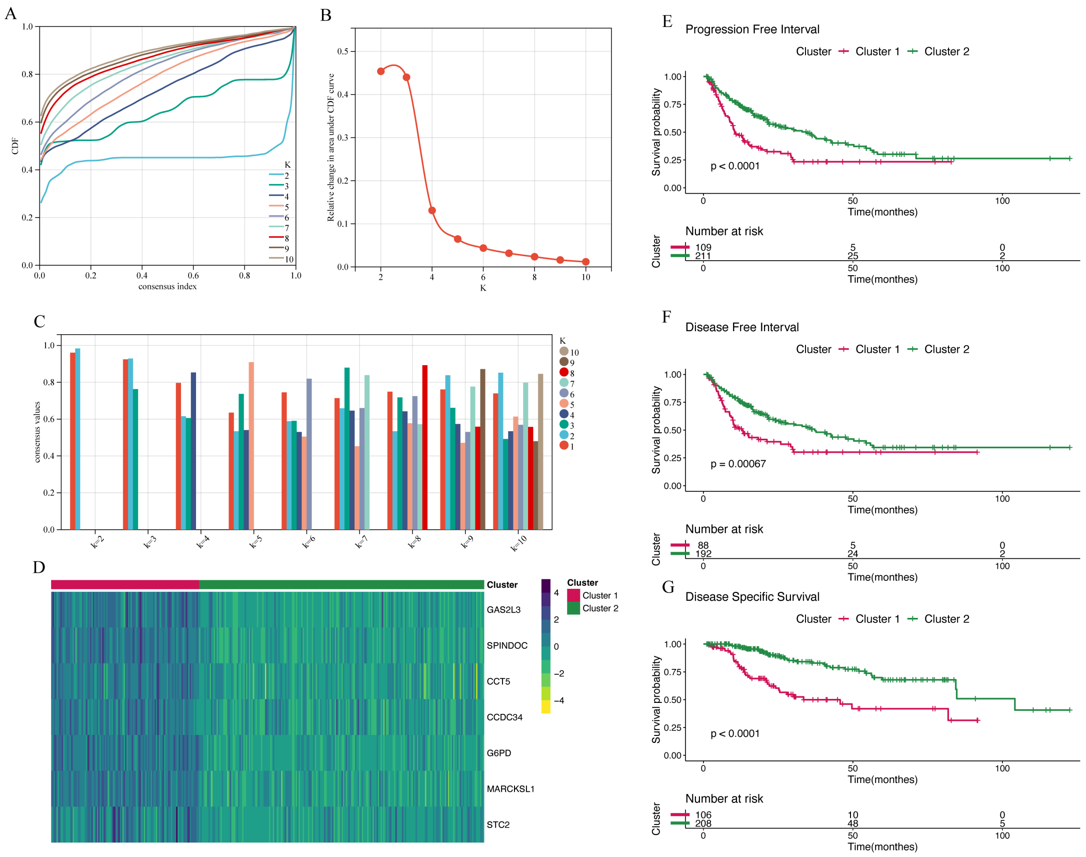

Figure S6. Construction of disulfidptosis-related clusters. (A) The area under the cumulative distribution function curve. (B) The Delta Area Plot. (C) Consensus values across different values of k. (D) A heatmap of 7 genes expression between two clusters. (E-G) Kaplan-Meier analysis for PFI, DFI, and DSS in different clusters.

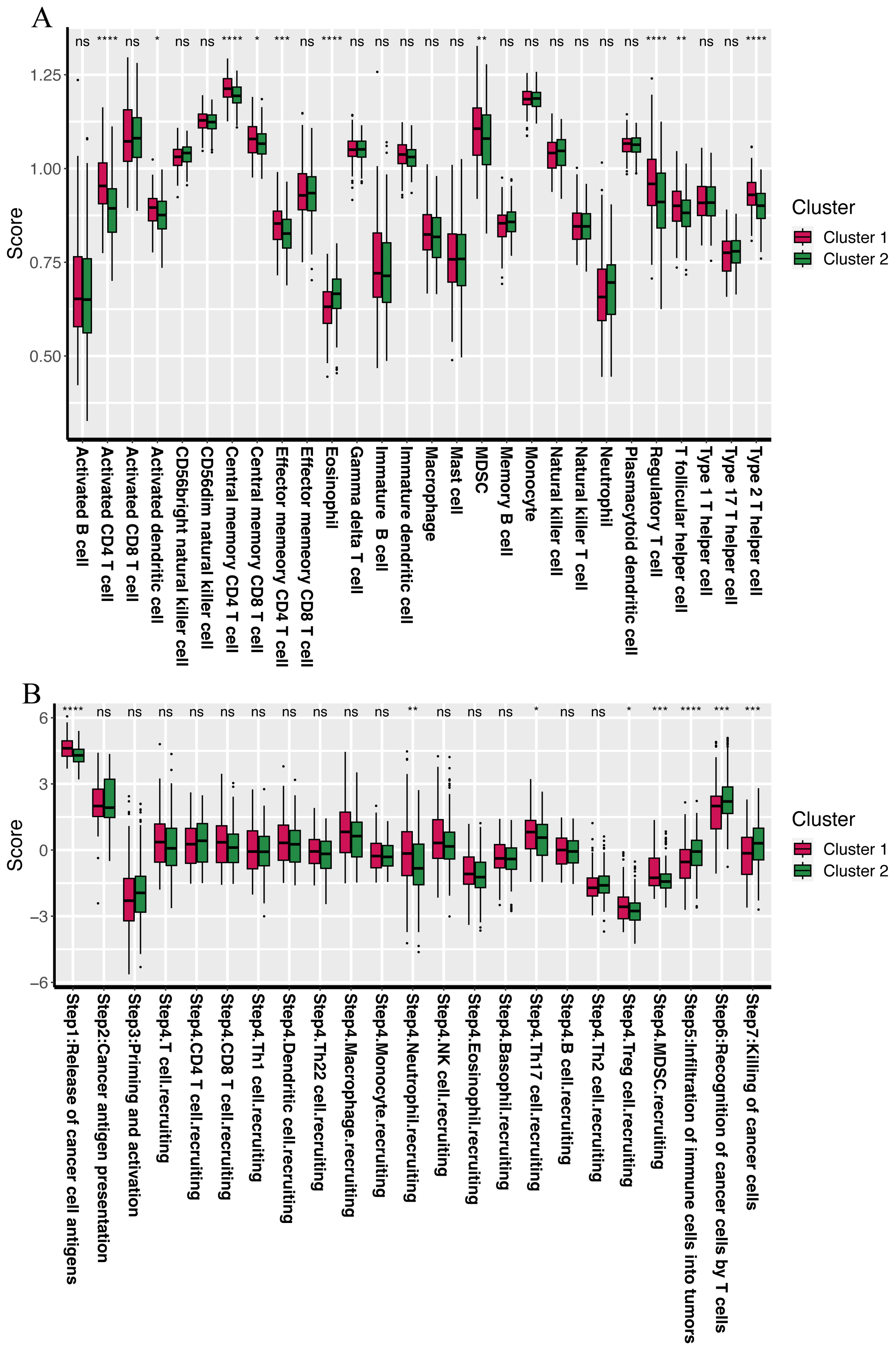

Figure S7. The relationship between the disulfidptosis-related clusters and the tumor microenvironment. (A) Comparison of the infiltration levels of 28 immune cell types between 2 disulfidptosis-related clusters. (B) Cancer-Immunity Cycle status

between 2 disulfidptosis-related clusters

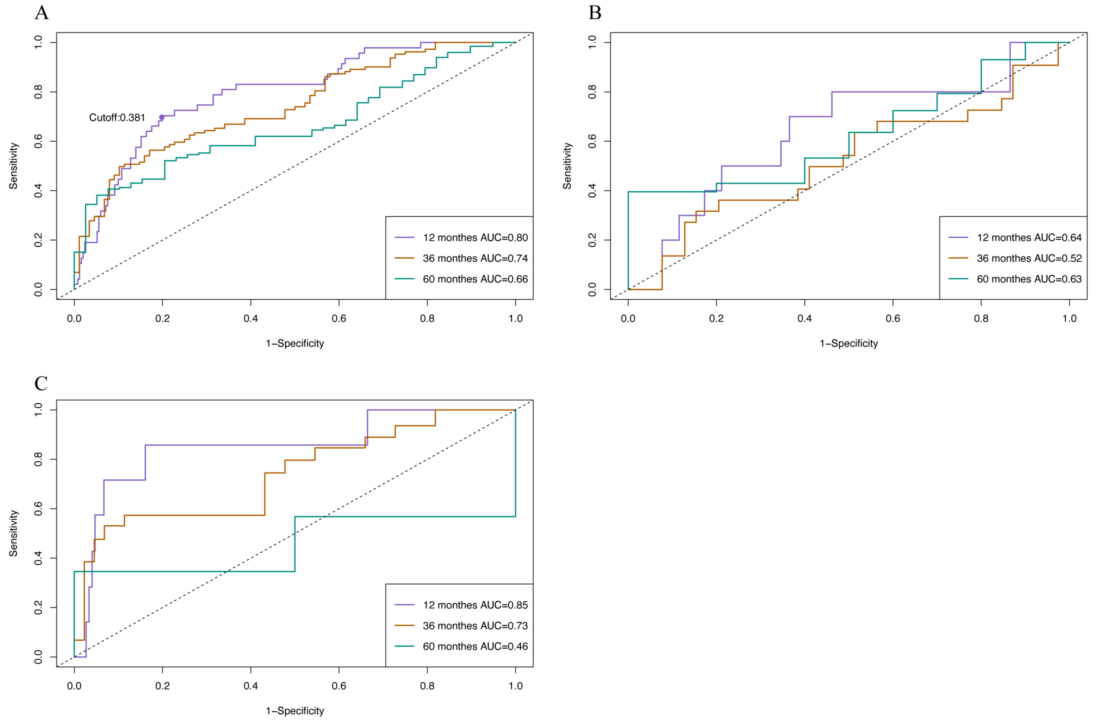

Figure S8. Time-dependent ROC analysis of the DR score in three cohorts. (A) Time-dependent ROC analysis of the DR score in TCGA-LIHC. (B) Time-dependent ROC analysis of the DR score in GSE116174. (C) Time-dependent ROC analysis of the DR score in LIRI-JP.
